# Optogenetic Control of Bacterial Expression by Red Light

**DOI:** 10.1021/acssynbio.2c00259

**Published:** 2022-08-23

**Authors:** Elina Multamäki, Andrés García de Fuentes, Oleksii Sieryi, Alexander Bykov, Uwe Gerken, Américo Tavares Ranzani, Jürgen Köhler, Igor Meglinski, Andreas Möglich, Heikki Takala

**Affiliations:** † Department of Anatomy, University of Helsinki, Helsinki 00014, Finland; ‡ Lehrstuhl für Biochemie, Photobiochemie, Universität Bayreuth, Bayreuth 95447, Germany; § Optoelectronics and Measurement Techniques, University of Oulu, Oulu 90014, Finland; ∥ Lehrstuhl für Spektroskopie weicher Materie, Universität Bayreuth, Bayreuth 95447, Germany; ⊥ College of Engineering and Physical Sciences, Aston University, Birmingham B4 7ET, U.K.; # Department of Biological and Environmental Science, Nanoscience Center, University of Jyvaskyla, Jyvaskyla 40014, Finland

**Keywords:** gene expression, optogenetics, phytochrome, sensory photoreceptor, signal transduction, two-component system

## Abstract

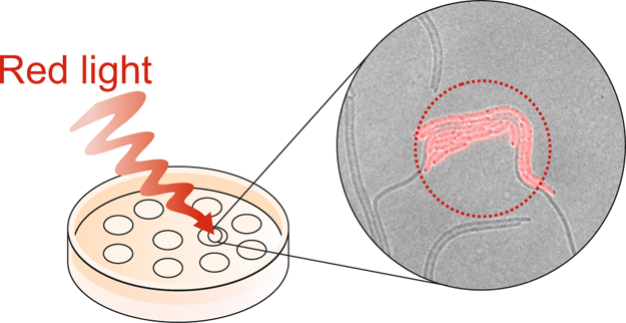

In optogenetics, as in nature, sensory photoreceptors serve to control cellular processes by light. Bacteriophytochrome (BphP) photoreceptors sense red and far-red light via a biliverdin chromophore and, in response, cycle between the spectroscopically, structurally, and functionally distinct Pr and Pfr states. BphPs commonly belong to two-component systems that control the phosphorylation of cognate response regulators and downstream gene expression through histidine kinase modules. We recently demonstrated that the paradigm BphP from *Deinococcus radiodurans* exclusively acts as a phosphatase but that its photosensory module can control the histidine kinase activity of homologous receptors. Here, we apply this insight to reprogram two widely used setups for bacterial gene expression from blue-light to red-light control. The resultant pREDusk and pREDawn systems allow gene expression to be regulated down and up, respectively, uniformly under red light by 100-fold or more. Both setups are realized as portable, single plasmids that encode all necessary components including the biliverdin-producing machinery. The triggering by red light affords high spatial resolution down to the single-cell level. As pREDusk and pREDawn respond sensitively to red light, they support multiplexing with optogenetic systems sensitive to other light colors. Owing to the superior tissue penetration of red light, the pREDawn system can be triggered at therapeutically safe light intensities through material layers, replicating the optical properties of the skin and skull. Given these advantages, pREDusk and pREDawn enable red-light-regulated expression for diverse use cases in bacteria.

## Introduction

The analysis of complex biological systems greatly benefits from innovative tools for the observation and perturbation of cellular events with precision in time and space, yet minimal invasiveness. Optogenetics meets these demands by genetically encoding photoreceptors to control the state and dynamics of target cells by light. Initially confined to the neurosciences and reliant on light-gated rhodopsin ion channels and pumps,^1−3^ optogenetics has transcended its beginnings and now allows the optical control of diverse cellular processes.^4−7^ Commensurate with its eminent relevance for life, gene expression has been a frequent target of optogenetics. In both prokaryotes and eukaryotes, light-dependent expression control is mostly achieved at the level of transcription initiation,^5,8−10^ but other control points have also been addressed by optogenetics.^11−13^

In bacteria, several setups for light-regulated gene expression rely on two-component signaling systems (TCSs). These systems are mainly found in prokaryotes and allow cells to process environmental signals into intracellular responses.^14^ In their canonical form, TCSs consist of a sensor histidine kinase (HK) that phosphorylates its cognate response regulator (RR) in a signal-dependent manner. HKs commonly exert elementary kinase and phosphatase activities.^15,16^ Depending on the presence of signal, one or the other activity predominates, and the HK enzyme is rendered a net kinase or net phosphatase. Several TCSs have previously been harnessed for the optogenetic control of gene expression in *Escherichia coli*,^8,17−20^ and more recently in *Bacillus subtilis*.^21^ For instance, the pDusk plasmid^18^ is based on YF1, an artificial HK with a light-oxygen-voltage (LOV) photosensory domain,^22^ and its response regulator FixJ.^23^ While in darkness, YF1 acts as a net kinase on FixJ, blue light converts it into a net phosphatase that efficiently removes phosphoryl groups from phospho-FixJ.^23,24^ Expression of target genes is thus lowered by around 15-fold under blue light compared to that in darkness. The pDawn plasmid derives from pDusk and harbors a gene-inversion cassette based on the λ phage cI repressor; blue light hence leads to an upregulation of target gene expression by several 100-fold.^18^ Tabor and colleagues pioneered and refined the optogenetic deployment of the CcaS/R TCS.^20^ At the heart of the system lies CcaS, a cyanobacteriochrome (CBCR)-coupled HK that responds to red and green light and phosphorylates the RR CcaR. CBCRs form a subclass of the phytochrome photoreceptor superfamily and covalently incorporate the reduced bilin phycocyanobilin (PCB) as their light-sensitive pigment. Since its inception, the CcaRS system has been iteratively optimized, and its present incarnation achieves several 100-fold upregulation of target gene expression under green light compared to that under red light.^25^

Notwithstanding the availability of several photoresponsive TCSs and other implements for light-regulated bacterial expression,^8,18,20,25−32^ a need exists for red-light-regulated optogenetic systems. Owing to much better tissue penetration of long wavelengths compared to short ones,^33^ such tools would enable studies in deep tissues and allow less harmful illumination.^34^ Sensitivity to red and far-red light is afforded by phytochrome photoreceptors which were originally discovered in plants^35^ but also occur in bacteria and fungi.^14^ Phytochromes bind linear tetrapyrrole (bilin) chromophores and cycle between the red-light-absorbing Pr state and the far-red light-absorbing Pfr state in response to red/far-red light irradiation.^36^ The chromophore is embedded within a GAF domain (cGMP phosphodiesterase-adenylate cyclase FhlA) which together with the PAS (Per-ARNT-Sim) and PHY (phytochrome-specific) domains is part of the photosensory module (PSM), also referred to as the photosensory core module.^37^ Bacterial phytochromes (BphPs) usually carry enzymatically active effector modules,^6^ with the majority belonging to TCSs that act as HKs and transduce signals to RR proteins.^38^ As demonstrated for the model BphP from *Deinococcus radiodurans*, light-induced structural changes in the PSM^39^ relay to the effector HK domain and thereby change its elementary kinase and phosphatase activities.^40,41^

The first bacterial setup for gene expression that responds to red and far-red (i.e., near-infrared) light was based on the chimeric phytochrome Cph8, which consists of the PSM of the cyanobacterial phytochrome Cph1 and the HK domain from *E. coli* EnvZ.^8^ Together with the *E. coli* RR OmpR, Cph8 mediated red-light-dependent optogenetic control of bacterial gene expression. However, the Cph8/OmpR TCS has several limitations that may complicate its application. First, being of cyanobacterial origin, Cph8 relies on the PCB chromophore that is specific to cyanobacteria but not to *E. coli*. The chromophore thus requires supplementation or endogenous production from heme via co-expression of both heme oxygenase (HO) and biliverdin reductase (PcyA).^42^ By contrast, BphPs use biliverdin (BV) which can be produced inside cells from heme through the action of HO alone. Second, Cph8 harnesses the endogenous EnvZ/OmpR TCS, thus potentially causing crosstalk and pleiotropic responses when applied in *E. coli*.

Against this backdrop, we assessed here whether the established pDusk and pDawn optogenetic circuits^18^ can be reprogrammed from blue-light control to red-light control. If successful, the utility of these widely used optogenetic implements would be much extended. By substituting the LOV photosensor module of YF1 for the PSM of the *D. radiodurans* BphP, we engineered the novel optogenetic tools pREDusk and pREDawn that respond to red and far-red light and allow spatiotemporally precise control of gene expression in *E. coli*. pREDusk exhibits more than 200-fold downregulation of gene expression under red light, thus greatly surpassing the regulatory response of the parental pDusk to blue light. By contrast, pREDawn activates target gene expression under red light by almost 100-fold. Owing to its high light sensitivity, pREDawn can be efficiently activated by red light at therapeutically safe illumination intensities through tissue phantoms with the optical properties of mouse skin and skull.

## Results

### Design of pREDusk and pREDawn

Setting out to engineer systems for red-light-dependent expression in bacteria, we derivatized the pDusk and pDawn plasmids, given that they afford pronounced regulatory responses^18^ and underpin numerous applications in optogenetics, biotechnology, and synthetic biology, see for example, refs (28), (43)–^48^. Both plasmids employ the blue-light-sensitive YF1 which originates from a fusion between the LOV photosensor of *B. subtilis* YtvA and the HK module of *Bradyrhizobium japonicum* FixL.^23^ To expedite plasmid derivatization, we put the red-fluorescent reporter gene *Ds*Red Express2, referred to as *Ds*Red in the following, under control of the FixK2 promoter which in pDusk is regulated by the YF1/FixJ TCS in a light-dependent manner.^18,49,50^

**Figure 1:**
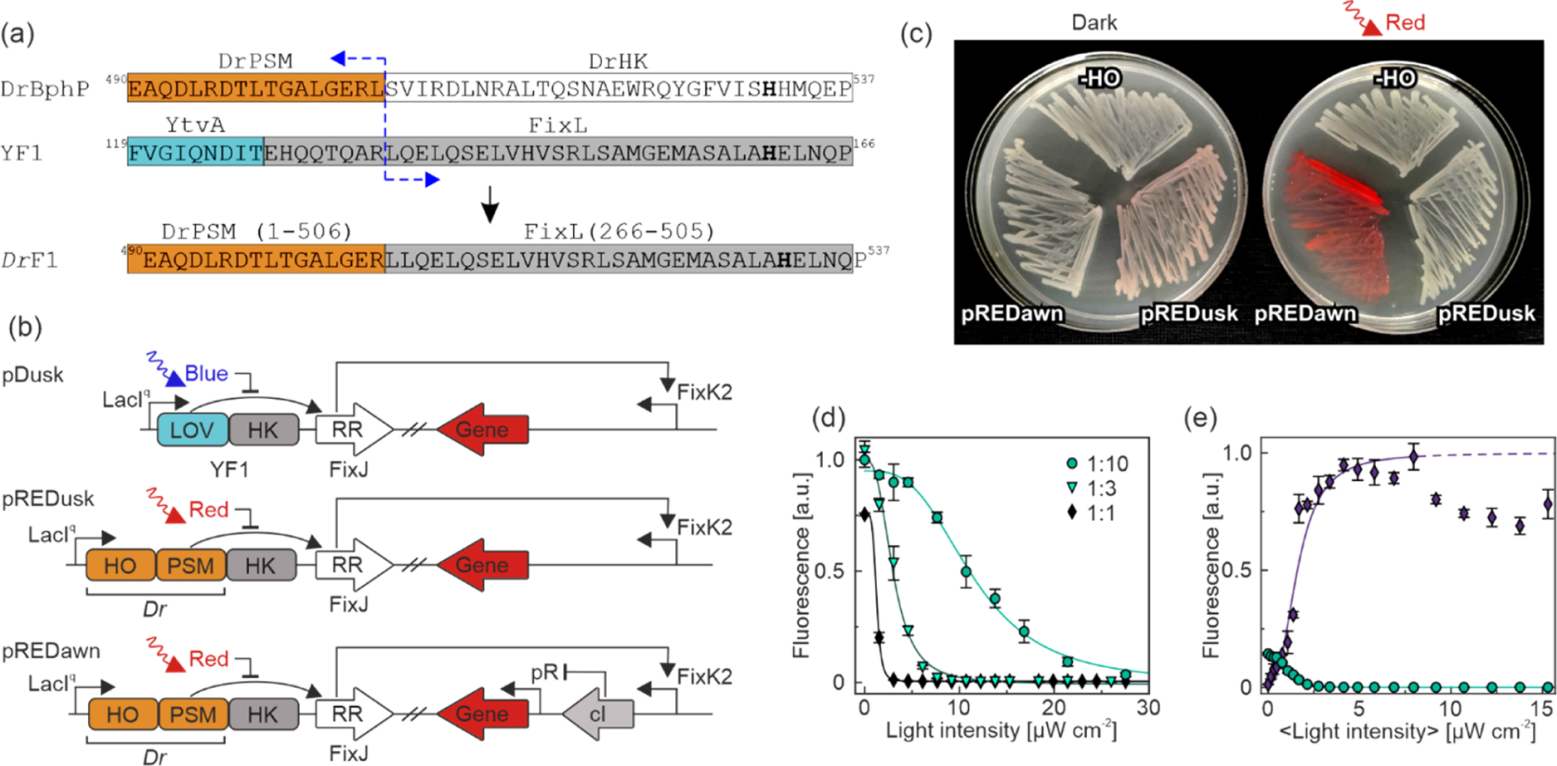
Architecture and function of the pREDusk and pREDawn expression systems. (a) Sequence alignment of the *Deinococcus radiodurans* phytochrome (*Dr*BphP) and YF1 informs the design of the *Dr*F1 chimeric histidine kinase. (b) In pREDusk, the blue-light-controlled light-oxygen-voltage (LOV) photosensor of the parental pDusk plasmid is replaced with heme oxygenase (HO) and the *Dr*BphP PSM. pREDawn is derived from pREDusk and harbors a gene-inversion cassette based on the λ phage cI repressor and its target promoter pR. (c) Expression of a *Ds*Red fluorescent reporter is diminished under red light in *E. coli* bacteria harboring pREDusk-*Ds*Red. *Vice versa*, pREDawn-*Ds*Red mediates red-light-activated *Ds*Red expression. If HO is excluded from pREDusk-*Ds*Red, no reporter expression is discernible in darkness or under red light. (d) Bacteria harboring the pREDusk-*Ds*Red plasmid were cultivated while being exposed to intermittent red light at varying intensities and at duty cycles of 1:1, 1:3, or 1:10. Following incubation, the *Ds*Red reporter fluorescence in the cultures was normalized by the optical density of the cultures at 600 nm. Plots are normalized to the maximum value obtained for the 1:10 duty cycle. (e) Using a 1:10 duty cycle, the response to red light of bacteria carrying either pREDusk-*Ds*Red (circles) or pREDawn-*Ds*Red (diamonds) was probed. Plots are normalized to the maximum value of the pREDawn-*Ds*Red setup. Data in (d and e) represent mean ± s.d. of three biological replicates.

To confer sensitivity to red and far-red light, we exchanged the LOV photosensor of YF1 in pDusk for the PSM of *D. radiodurans* BphP (*Dr*PSM). This choice was dictated by the ample knowledge on the photochemistry, structure, and conformational dynamics of *Dr*BphP, which arguably render this receptor the best-characterized of all phytochromes.^39,51−53^ Moreover, in the past, the *Dr*PSM served to bestow light sensitivity on diverse effectors including nucleotidyl cyclases,^54−56^ phosphodiesterases,^57,58^ and receptor tyrosine kinases.^59^ Lastly, we have recently demonstrated that the *Dr*PSM can principally control both the kinase and phosphate activities of HK effectors, although *Dr*BphP itself exclusively acts as a phosphatase.^41^ Guided by an alignment of the HK moieties of YF1/FixL and *Dr*BphP (Figure 1a), we selected residues 1–506 of *Dr*BphP, encompassing its PSM and part of the linker to the effector, for the subsequent design. This *Dr*PSM length matched that within the phosphodiesterase fusion^57,58^ but differed by a few residues from the nucleotidyl cyclase (amino acids 1–510)^54^ and receptor tyrosine kinase fusions (amino acids 1–504).^59^ The *Dr*PSM (1–506) was connected to residues 136–380 of YF1, corresponding to residues 266–505 of the original *B. japonicum* FixL HK. We refer to the resultant chimeric HK as *Dr*F1 in the following. Within the plasmid, *Dr*F1 is thus combined into one operon with the RR FixJ from *B. japonicum* and placed under control of the constitutive *lacI*^*q*^ promoter (see Figure 1b).

BphP receptors require the covalent incorporation of biliverdin to respond to light. Although the genomes of at least certain *E. coli* strains encode HOs that are capable of forming BV,^60^ the functional expression and application of BphP proteins in bacteria are routinely supported by co-expression of a HO enzyme.^42,61^ We selected the HO from *D. radiodurans* (*Dr*HO) to this end, given that in the *D. radiodurans* genome its gene directly precedes the BphP gene within the same operon. We thus replicated the genomic operon architecture, placed *Dr*HO immediately upstream of the *Dr*PSM, and thereby furnished a tricistronic operon (*Dr*HO, *Dr*PSM-FixL, and *Bj*FixJ), all under control of the *lacI*^*q*^ promoter (Figure 1b). Upon transformation of the resultant plasmid into *E. coli*, the bacteria were plated on LB agar and incubated overnight at 37 °C either in darkness or under constant red light (660 nm, 100 μW cm^–2^). Visual inspection revealed discernible expression of the *Ds*Red reporter gene in darkness but not under red light (Figure 1c), and we hence named the plasmid pREDusk (Table 1). The data indicate that *Dr*F1 acts as a red-light-repressed histidine kinase, analogous to the parental YF1 which exhibited reduced net kinase activity under blue light.^18,23^ As a control, we also assessed the activity and red-light responses of a plasmid variant lacking the *Dr*HO gene. Bacteria harboring this plasmid were colorless both in darkness and under red light (Figure 1c), indicating that the HO is indeed required for sufficient intracellular BV production and intact light responses.

**Table 1:** Constructs Generated in This Study

	construct	resistance	ori^a^	reporter gene	addgene ID
1	pREDusk	KanR	ColE1	*Ds*Red	
2	pREDawn	KanR	ColE1	*Ds*Red	
3	pREDusk-MCS	KanR	ColE1	MCS^b^	188970
4	pREDawn-MCS	KanR	ColE1	MCS	188971
5	pREDusk-StrR	StrR	CloDF13	*Ds*Red	
6	pREDawn-StrR	StrR	CloDF13	*Ds*Red	
7	pREDusk-StrR-MCS	StrR	CloDF13	MCS	188972
8	pREDawn-StrR-MCS	StrR	CloDF13	MCS	188979
9	pREDusk-AmpR	AmpR	ColE1	*Ds*Red	
10	pREDawn-AmpR	AmpR	ColE1	*Ds*Red	
11	pREDusk-AmpR-MCS	AmpR	ColE1	MCS	188974
12	pREDawn-AmpR-MCS	AmpR	ColE1	MCS	188978
13	pREDusk-YPet	KanR	ColE1	YPet	
14	pREDawn-YPet	KanR	ColE1	YPet	

a Origin of replication.

b Multiple cloning site.

We further generated the pREDawn plasmid with inverted signal polarity by inserting a gene cassette encoding the λ phage repressor cI and the λ promoter pR, as in the original pDawn system.^18^ Doing so puts cI expression under the control of the FixK2 promoter and that of the *Ds*Red reporter gene under the pR promoter (Figure 1b). Following plasmid transformation, plating, and overnight incubation, strong *Ds*Red expression occurred under red light but not in darkness (Figure 1c). Notably, under inducing conditions (i.e., darkness for pREDusk and red light for pREDawn), *Ds*Red expression was stronger for pREDawn-*Ds*Red than for pREDusk-*Ds*Red, consistent with the original pDusk/pDawn systems and arguably reflecting the relative strengths of the FixK2 and pR promoters.

To facilitate the use of pREDusk and pREDawn, we constructed plasmids in which the *Ds*Red reporter is replaced by a multiple cloning site (MCS) and deposited them with Addgene. To extend the toolkit further, we also generated several alternative pREDusk and pREDawn systems with modified origins of replication (ori) and antibiotic resistances (Table 1). The derivative variants maintained similar light responses as the original pREDusk and pREDawn (Figure S1) and can be combined with other vectors, for instance, for spectral multiplexing and co-expression experiments.

### Red-Light Responses of pREDusk and pREDawn

Next, we assessed the dose dependence of the two red-light-regulated gene expression systems by growing *E. coli* cultures bearing pREDusk-*Ds*Red or pREDawn-*Ds*Red in a 96-well microtiter plate (MTP) format. During growth, individual wells were illuminated from below with a programmable matrix of light-emitting diodes (LED) at a wavelength of (624 ± 8) nm.^62^ Following overnight incubation at 37 °C, the optical density at 600 nm (OD_600_) and the *Ds*Red fluorescence of the cultures were measured. Notably, the *Ds*Red fluorescence excitation spectrum has minimal overlap with the emission spectrum of the red LED used for optogenetic stimulation (Figure S5). Hence, the *Ds*Red protein will not be optically excited efficiently during cell growth and illumination. Once formed inside the bacteria, the *Ds*Red protein is highly stable, and its fluorescence persists over many hours (Figure S2a). *Ds*Red fluorescence readings were normalized by OD_600_ and corrected for background fluorescence. At constant illumination, the pREDusk-/pREDawn-*Ds*Red systems were already triggered to substantial extent at an intensity of 1.5 μW cm^–2^, the lowest setting of the programmable LED matrix. These findings qualitatively indicate high light sensitivity of the two systems. To enable probing of the dose–response relationship, we assessed pulsed illumination at different duty cycles in the pREDusk-*Ds*Red system, thus reducing the average light intensity applied during the experiment (Figure 1d). At all tested duty cycles (1:1, 1:2, 1:3, 1:5, and 1:10), the response of the pREDusk-*Ds*Red system depended on the average light intensity but not on the timing of intermittent illumination (Figure S2b). All subsequent experiments were thus performed at a 1:10 duty cycle, meaning 20 s of illumination, followed by 180 s darkness. Under these conditions, the illumination did not notably affect the growth of the bacteria (Figure S3). In the following, we use angle brackets to denote light intensities averaged over the duty cycle.

In the pREDusk-*Ds*Red system (Figure 1e), *Ds*Red expression decreased monotonically with applied red-light intensity <I> by around 250-fold with a half-maximal red-light dose (I_50_) of (1.3 ± 0.4) μW cm^–2^. Interestingly, the pREDusk plasmid thus achieved a more stringent response to light than the parental pDusk plasmid, where gene expression was downregulated by a mere 15-fold under blue light.^18^ In the case of pREDawn-*Ds*Red (Figure 1e), reporter expression increased with red light with an I_50_ of (1.5 ± 0.2) μW cm^–2^ by up to around 77-fold at 8 μW cm^–2^. At yet higher light intensities, a drop of *Ds*Red fluorescence by around 20% occurred. Consistent with the initial assessment of the two plasmids (see Figure 1c), the maximum expression levels driven by pREDawn-*Ds*Red under red light were about 7.4-fold higher than those for pREDusk-*Ds*Red in darkness. By contrast, the basal expression level in pREDawn-*Ds*Red (in darkness) was around 20-fold higher than the basal level for pREDusk (under red light). The elevated basal expression in pREDawn-*Ds*Red accounts for the reduced regulatory response to light compared to pDawn-*Ds*Red which exhibited more than 400-fold upregulation of expression under blue light. For both pREDusk-*Ds*Red and pREDawn-*Ds*Red, the dose–response data yielded Hill coefficients above unity, which indicates a cooperative response to illumination. While at present the molecular origin remains unclear, cooperativity may at least in part be attributed to the homodimeric state of conventional HKs.^16,23,63^ This notion is supported by findings on YF1, which shares with *Dr*F1 the same histidine kinase effector.^23^ The two LOV photosensor units of the homodimeric YF1 cooperatively regulated the kinase output of the receptor. The derivative pREDusk and pREDawn variants with altered ori and resistance marker exhibited light responses qualitatively similar to the above pREDusk-*Ds*Red and pREDawn-*Ds*Red with kanamycin resistance markers (Figure S4). In the case of pREDawn with an ampicillin resistance marker, a more pronounced drop of *Ds*Red expression at high red-light intensities was observed.

**Figure 2:**
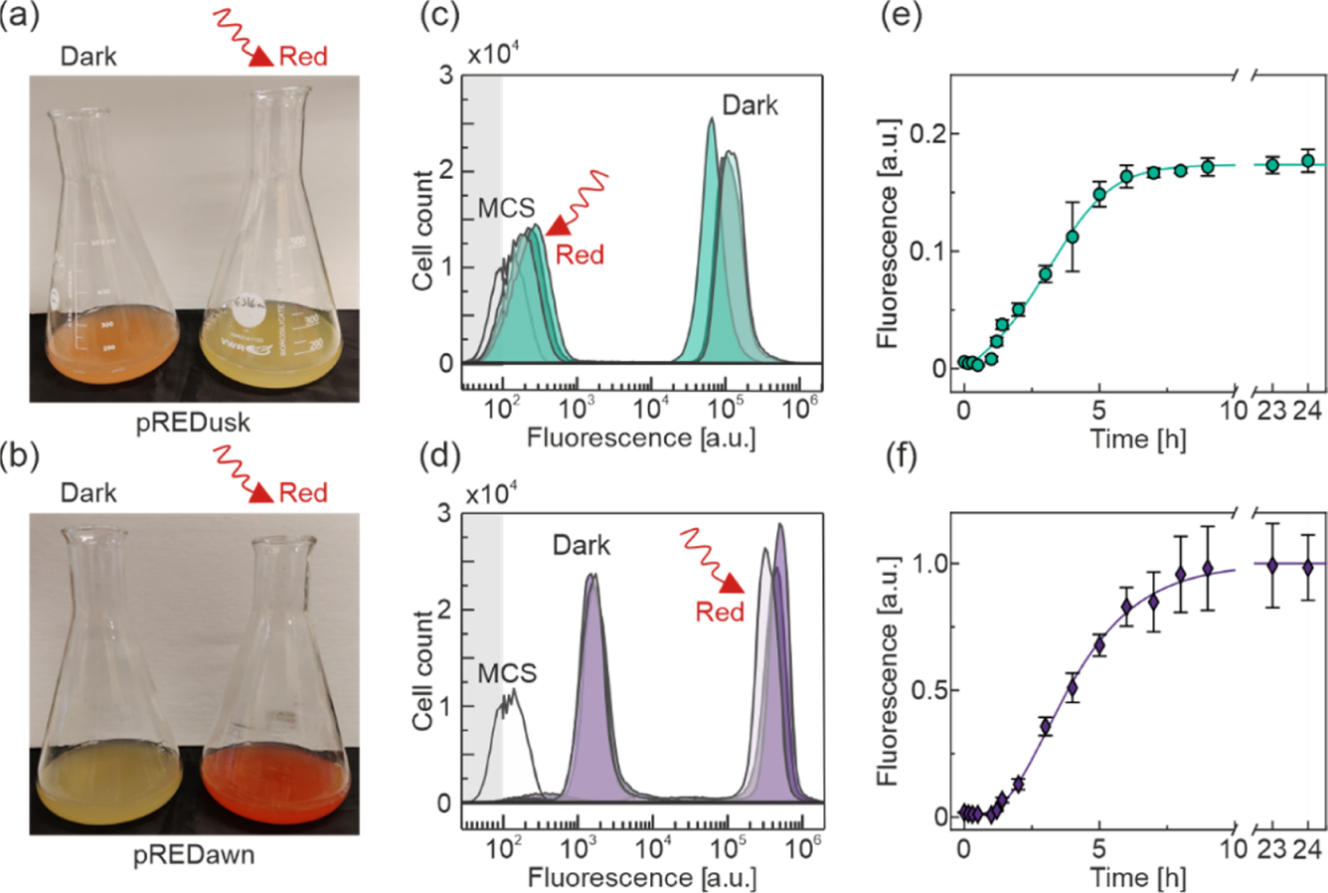
Preparative expression of *Ds*Red by pREDusk and pREDawn. (a) *E. coli* cells bearing either pREDusk-*Ds*Red or (b) pREDawn-*Ds*Red were cultured in 100 mL scale in darkness and under red light, respectively. The expression of the *Ds*Red reporter is readily visible as a color change in the cultures. (c) Flow cytometry analysis of the pREDusk-*Ds*Red cultures shown in (a). In darkness, the fluorescence peaked at 10^4.8^ arbitrary units (a.u), but under red light the mean fluorescence amounted to 10^2.1^ a.u., closely similar to an empty vector control. The fluorescence values above 10^2^ a.u. are plotted in a logarithmic scale and values under 10^2^ are presented in a linear scale, as indicated by gray shading. (d) Flow cytometry analysis of the cultures shown in (b). For pREDawn-*Ds*Red, the mean fluorescence in darkness was at 10^3.0^ a.u. compared to 10^5.5^ a.u. under red light. At least 100,000 cells were collected for each sample, and three representative runs are shown. (e) 100 mL cultures harboring pREDusk-*Ds*Red were grown under non-inducing conditions (i.e., 100 μW cm^–2^ red light) to an optical density at 600 nm of around 0.4. Upon transfer to inducing conditions (i.e., darkness), the *Ds*Red reporter fluorescence increased sigmoidally with a half-maximal time *t*_50_ of (2.8 ± 0.2) h. (f) pREDawn-*Ds*Red cultures were analyzed likewise except that they were first grown under non-inducing dark conditions before being transferred to inducing red light. The half-maximal response was at (4.1 ± 0.6) h.

To assess how uniformly individual bacteria respond to light stimuli, we complemented the above ensemble experiments by single-cell fluorescence measurements via flow cytometry (Figure 2). We grew bacteria bearing the pREDusk-*Ds*Red or pREDawn-*Ds*Red systems in 100 mL scale for 20 h at 37 °C in darkness or under constant red light (660 nm, 100 μW cm^–2^) (Figure 2a,b). *Ds*Red fluorescence was measured in single cells by flow cytometry at 561 nm excitation and (586 ± 20) nm emission. Under non-inducing conditions (red light), the bacteria with the pREDusk-*Ds*Red circuit exhibited a mean fluorescence of 10^2.1^ relative fluorescence units (RFU), which is highly similar to the pREDusk-MCS background control at 10^2.3^ RFU (Figure 2c). In agreement with the ensemble measurements, cells harboring pREDusk showed strongly enhanced mean fluorescence of 10^4.8^ RFU when cultivated in darkness, which corresponds to a 470-fold upregulation compared to incubation under red light. Remarkably, the entire population shifted homogeneously from low fluorescence under non-inducing conditions to high fluorescence under inducing conditions; and no minor or unresponsive cell population was visible. For pREDawn-*Ds*Red, the mean fluorescence amounted to 10^3.0^ RFU under non-inducing dark conditions (Figure 2d). Red light prompted the mean fluorescence to increase by around 290-fold to 10^5.5^ RFU. While a major part of the clonal population (90–97%) shifted to high fluorescence intensities upon red-light induction, a minor population of cells exhibited low fluorescence levels corresponding to those of the background control. The second population may be indicative of plasmid loss at prolonged incubation under conditions of strong induction.

We next assessed how rapidly the pREDusk and pREDawn systems respond to changes in illumination (Figure 2e,f). For this purpose, 100 mL cultures of bacteria carrying pREDusk-*Ds*Red or pREDawn-*Ds*Red were grown to an OD_600_ of 0.5 under non-inducing conditions and were then transferred to inducing conditions, that is, darkness for pREDusk-*Ds*Red and 660 nm light (100 μW cm^–2^) for pREDawn-*Ds*Red. Aliquots were taken at the time of induction and for up to 24 h afterward. Immediately upon sampling, cell growth and translation of the samples were halted by addition of high concentrations of chloramphenicol and tetracycline.^18^ After allowing for *Ds*Red maturation,^49^ the fluorescence of the aliquots was determined and divided by OD_600_. The normalized fluorescence readings for both pREDusk-*Ds*Red (Figure 2e) and pREDawn-*Ds*Red (Figure 2f) increased sigmoidally over time. Already at 60 and 80 min after induction, an upregulation of reporter fluorescence was observed for pREDusk and pREDawn, respectively. We evaluated the data according to a logistic function to determine the time *t*_50_, at which the systems are half-maximally induced. The *t*_50_ values for pREDusk and pREDawn were (2.8 ± 0.2) h and (4.1 ± 0.6) h, respectively.

### Far-Red Light Counteracts Red Light

**Figure 3:**
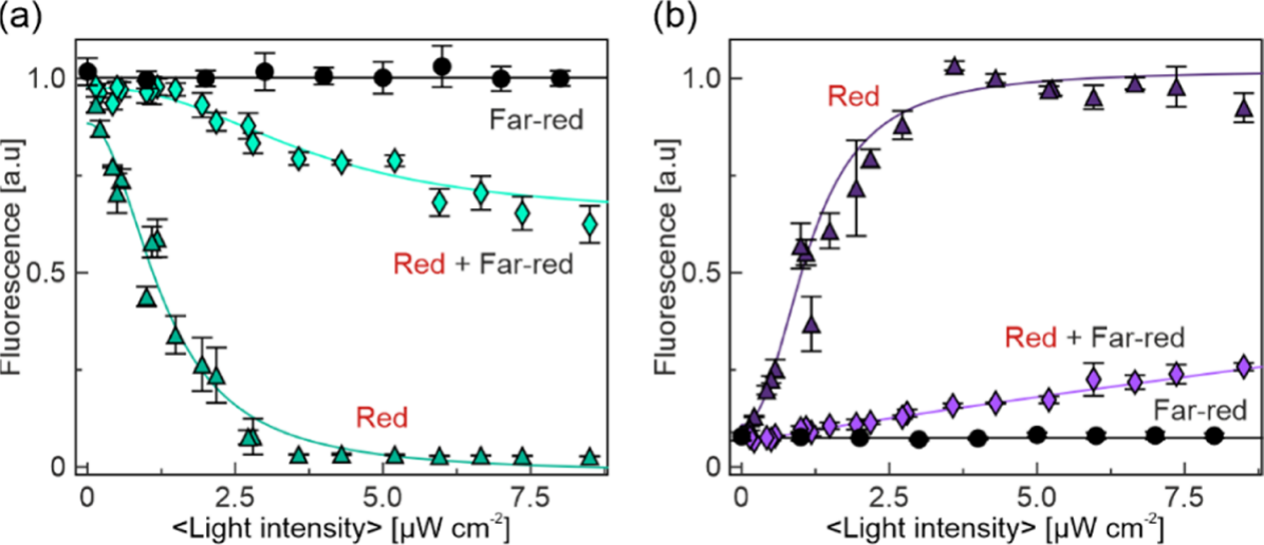
Far-red light counteracts the red light-induced effects. (a) Bacteria harboring pREDusk-*Ds*Red were cultivated at varying intensities of red light (triangles) or far-red light (circles). In another experiment (diamonds), bacteria were grown while being simultaneously illuminated with 60 μW cm^–2^ far-red light and varying intensities of red light. (b) As in (a) but for pREDawn-*Ds*Red. See Figure S5a for the emission spectra of the light sources.

Given that phytochromes photochromically interconvert between their Pr and Pfr states, we next assessed whether the pREDusk and pREDawn systems also respond to far-red light in addition to red light (Figure 3). We grew bacteria harboring the pREDusk-*Ds*Red and pREDawn-*Ds*Red plasmids in MTP format and illuminated them with a customized, programmable matrix of LEDs emitting at (655 ± 10) nm and (850 ± 21) nm, respectively.^56^ When far-red light was applied at an average intensity of 60 μW cm^–2^ simultaneously with red light, the responses of both pREDusk and pREDawn were greatly attenuated. At the highest tested average red-light intensity of 8.5 μW cm^–2^, the responses were only 25% of the maximal extents in the absence of far-red light. Even at the highest achievable intensity of far-red light used in these experiments, triggering by red light could not be fully suppressed. We note, however, that the emission of the infrared LEDs with a maximum at 850 nm only partially overlaps with the Pfr-state absorbance of the *Dr*PSM (Figure S5a). Use of infrared LEDs emitting at shorter wavelengths may enhance the suppression efficiency. Nonetheless, the present observations resemble findings on certain other optogenetic setups and could indicate sluggish or incomplete reversion by far-red light.^54,56−58^

### Spatially Resolved Gene Expression

**Figure 4:**
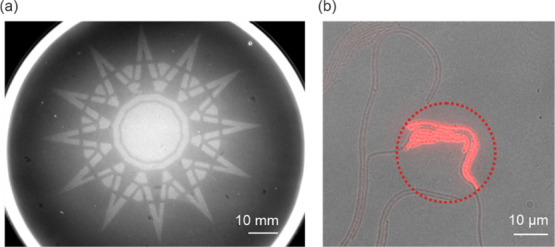
Spatially resolved activation of pREDawn. (a) Bacteria harboring pREDawn-*Ds*Red were embedded in agar. After projecting a photomask on the bacterial lawn and overnight incubation, *Ds*Red reporter fluorescence (white coloring) was detected in the illuminated areas. Brightness and contrast were adjusted uniformly across the entire image. The scale bar denotes 10 mm. (b) Individual bacteria containing pREDawn-*Ds*Red were illuminated for 1 h with 640 nm light focused on a circular patch (dotted circle). Following incubation in darkness for 2.5 h, *Ds*Red fluorescence was recorded. The image shows an overlay of the bright-field and fluorescence channels, with the scale bar indicating 10 μm. Brightness and contrast were adjusted uniformly across the entire field of view.

A specific advantage of optogenetically triggered systems over conventional, chemically inducible ones is the degree of spatial control afforded. To demonstrate this aspect, we prepared a lawn of bacteria harboring the pREDawn-*Ds*Red plasmid.^8,64,65^ Using a 640 nm laser at 200 μW cm^–2^ emitted power, we projected an image on the lawn for 5 min. After overnight incubation at 37 °C in darkness, the bacteria were monitored for fluorescence using a conventional transilluminator. The bacterial lawn faithfully reproduced the projected image with high accuracy (Figure 4a), indicating that the pREDawn system, and by extension, also pREDusk, can control gene expression with high spatial resolution.

To study spatial resolution at a single-cell scale (Figure 4b), *E. coli* cells carrying pREDawn-*Ds*Red were grown under non-inducing (dark) conditions and transferred to 3 cm glass dishes. Cells were then globally illuminated with 780 nm far-red light. Afterward, red light (640 nm) was focused onto a circular patch with ∼40 μm diameter for 1 h, followed by incubation in darkness, during which time the bacteria divided several times. After incubation, fluorescence images were collected to visualize the increase in *Ds*Red expression within bacteria inside the illuminated area. Change in *Ds*Red expression was clearly visible 3.5 h after the start of red-light exposure, with around 30-fold increase in *Ds*Red fluorescence in the illuminated areas compared to non-illuminated areas.

### Multimodal Optogenetic Control of Bacterial Gene Expression

The above experiments revealed that pREDusk and pREDawn mediate pronounced gene-regulatory responses to red light. We next addressed whether these systems can be combined with other optogenetic setups that respond to blue rather than red light. If so, multimodal optogenetic control would enable the separate and sequential triggering of distinct processes by light. Prospectively, such approaches might benefit applications of optogenetics in biotechnology and metabolic engineering, for instance, the regulation of multi-step enzyme cascades. Although multimodal optogenetic control was established in bacteria before,^20,28^ it often required the elaborate optimization of plasmids and strains. Against this backdrop, we cloned the YPet fluorescent protein into pREDusk and pREDawn and combined the resultant plasmids with the pCrespusculo-*Ds*Red and pAurora-*Ds*Red systems that are based on the RNA-binding LOV receptor PAL^11^ and respond to blue light.^66^ Briefly, pCrepusculo encodes PAL, which upon activation by blue light specifically binds to a short RNA aptamer. Said aptamer is embedded near the ribosome-binding site of an mRNA encoding *Ds*Red. Blue light thus prompts PAL binding and downregulation of fluorescence. The pAurora plasmid derives from pCrepusculo and resorts to the λ phage cI repressor to invert the system response to light, conceptually similar to pDawn and pREDawn.

**Figure 5:**
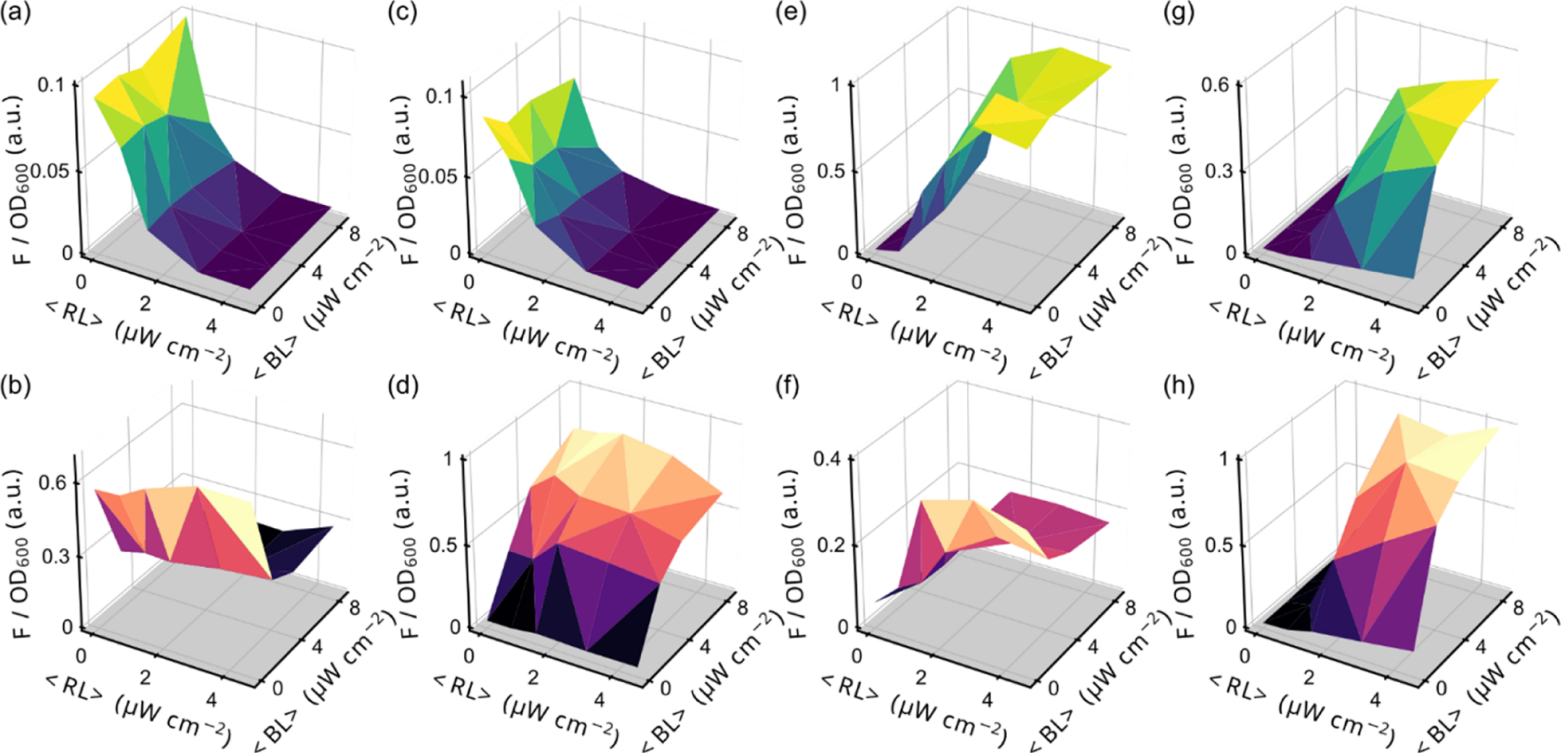
Multimodal optogenetic use of pREDusk/pREDawn combined with pCrepusculo/pAurora. (a–h) Bacteria harboring either pREDusk-YPet or pREDawn-YPet were cotransformed with either pCrepusculo-*Ds*Red or pAurora-*Ds*Red. (a–b) Combination of pREDusk-YPet and pCrepusculo-*Ds*Red. (c–d) Combination of pREDusk-YPet and pAurora-*Ds*Red. (e–f) Combination of pREDawn-YPet and pCrepusculo-*Ds*Red. (g–h) Combination of pREDawn-YPet and pAurora-*Ds*Red. Bacterial cultures were grown and subjected to different intensities of blue and red light (Figure S5b). Following growth, the *Ds*Red and YPet fluorescence readings were normalized by the optical density of the cultures at 600 nm (OD_600_) and corrected for background fluorescence. The top panels (a, c, e, and g) show YPet fluorescence, driven from either pREDusk or pREDawn, and the bottom panels (b, d, f, and h) report *Ds*Red fluorescence, governed by the pCrepusculo or pAurora plasmids. The *x* and *y* axes denote the average red-light and blue-light intensities, <RL> and <BL>, respectively, applied during the experiment. All data represent mean ± s.d. of three biological replicates.

We studied the response to blue and red light of bacteria that carry either pREDusk-YPet or pREDawn-YPet in combination with either pCrepusculo-*Ds*Red or pAurora-*Ds*Red (Figures 5 and S5b). When combined with either the pCrepusculo or pAurora plasmids, pREDusk-YPet gave rise to YPet fluorescence that diminished with increasing red-light intensity but did not depend on blue light (Figure 5a,c). Contrarily, the *Ds*Red fluorescence in these combined systems either decreased or increased with blue light for pCrepusculo-*Ds*Red and pAurora-*Ds*Red, respectively, but was unaffected by red light (Figure 5b,d). In the presence of pCrepusculo-*Ds*Red, pREDawn drove strong YPet expression under red light but was not triggered by blue light (Figure 5e). Whereas *Ds*Red fluorescence in this configuration was repressed by blue light as expected, additional repression occurred in the absence of red light (Figure 5f). We tentatively ascribe the unexpected red-light response of the pCrepusculo-YPet system to the presence of the λ cI repressor that forms part of the pREDawn circuit.

We finally assessed the combination of pREDawn-YPet and pAurora-*Ds*Red, both of which share the λ cI repressor to invert system response (Figure 5g,h). As expected, the two systems no longer responded independently to their respective light color but interacted. Both YPet and *Ds*Red fluorescence were synergistically upregulated by the simultaneous application of blue and red light, whereas each light color separately only produced a much weaker response. Put another way, the pREDawn and pAurora systems thus jointly established a logical AND gate. Importantly, both plasmids were used ‘out-of-the-box’, requiring no modification to either vector.

### Activation of Gene Expression through Tissue Phantoms

Sensitivity to red light not only facilitates multimodal optogenetics but also benefits applications in multicellular organisms, given that within the near-UV to near-IR region, long wavelengths penetrate biological tissues more readily than shorter ones.^33^ Although pREDusk and pREDawn are designed primarily for use in prokaryotes, there is scope for in vivo optogenetic applications inside animals. As a case in point, Cui et al. recently employed the pDawn setup to trigger gene expression in bacteria within the intestinal tract of mice.^34^ To overcome the limited penetration depth of blue light, upconverting nanoparticles were utilized to enable the stimulation of pDawn with near-infrared light.

**Figure 6:**
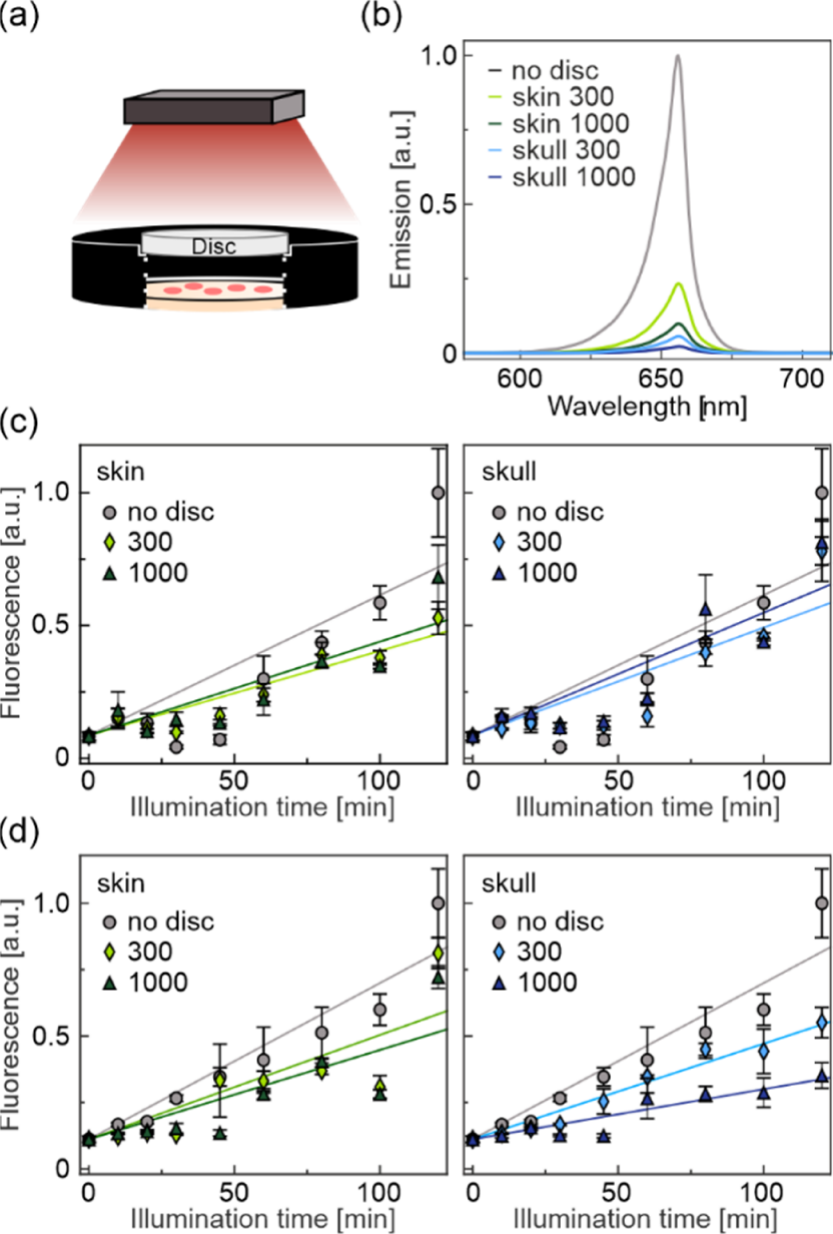
Activation of gene expression through tissue phantoms. (a) Schematic of the experimental setup. Bacterial colonies harboring the pREDawn-*Ds*Red plasmid were grown on an agar plate and exposed to 660 nm light. Phantom discs mimicking the optical properties of the mouse skin or skull were interposed in the light path. (b) Emission spectrum of the 660 nm LED light source without (no disc) or with one of the phantoms (skin or skull, 300 μm or 1000 μm) in the light path. (c–d) pREDawn-*Ds*Red activation through phantoms corresponding to the skin or skull tissue. After light exposure for up to 2 h, bacterial cultures were incubated overnight in darkness to allow for *Ds*Red expression and maturation. The applied light intensity was (c) 50 and (d) 5 μW cm^–2^. Fluorescence of the cultures was determined and normalized to the cell density. Data represent mean ± s.d. of three biological replicates. Also see Table S1 for details on the relative increase rates of fluorescence presented in (c and d).

To gauge the principal feasibility of such applications, we assessed to which extent pREDawn can be activated through biological tissues (Figure 6). For this purpose, we manufactured disc-shaped tissue phantoms that mimic the optical properties of 300 and 1000 μm thick mouse skin or skull at wavelengths between 600 and 900 nm (Figure 6a). Depending on the thickness and material, the phantoms attenuated the 660 nm light used for pREDawn activation by between 8- and 100-fold (Figure 6b). To probe whether the attenuated light sufficed for optogenetic activation, we grew bacteria carrying pREDawn-*Ds*Red on agar plates and illuminated them with red light (50 μW cm^–2^) for up to 2 h without or with one of the phantoms in the light path. Following overnight incubation to allow *Ds*Red expression and maturation, the fluorescence of the bacteria was determined. Without any phantom in the light path, the *Ds*Red fluorescence increased linearly with illumination time by up to around 10-fold after 2 h light exposure compared to bacteria not exposed to any light (Figure 6c and Table S1). Strikingly, fluorescence responses of similar extents were observed with the tissue phantoms in the light path (up to between 7- and 9-fold-increased fluorescence). Though light was greatly attenuated by the phantoms, the remaining intensity evidently sufficed to trigger pREDawn nearly fully. We hence repeated the experiment at a light intensity of 5 μW cm^–2^ (Figure 6d). While bacteria not shielded by any phantom showed *Ds*Red expression to similar extents as at 50 μW cm^–2^, reduced expression resulted when phantoms were interposed in the light path. For instance, upon 2 h illumination at 5 μW cm^–2^, the fluorescence increased by around 10-fold when no phantom was present but by only 3-fold for the opaquest phantom, corresponding to mouse skull of 1,000 μm thickness. However, even at the lower light intensity, clear-cut responses were observed for all tissue phantoms. Taken together, our findings suggest that pREDawn can be toggled inside biological tissues with moderate light intensities, owing to the high sensitivity of the system (see Figure 1e). Notably, the maximum area intensity of 50 μW cm^–2^ in our experiments is around 200-fold less than that commonly used for red-light therapy.^67^ Taken together, our findings indicate that optogenetic activation of pREDawn can be achieved through biological tissues at a comparatively moderate red-light exposure.

## Discussion

### Applications in Optogenetics and Synthetic Biology

Notwithstanding the advent of highly diverse and ingenious optogenetic strategies over the past decade,^5−7^ the use of light-regulated gene expression remains widespread, particularly in bacteria. The optogenetic control of gene expression offers a robust and adaptable means of altering bacterial physiology and metabolism by light, provided a temporal resolution in the minute range or above suffices for the desired use. Numerous applications in optogenetics, biotechnology, and synthetic biology bear out the versatility of light-regulated gene expression. For instance, optogenetics has been employed to trigger the onset of bioproduction processes,^43^ to spatially pattern gene expression and thereby template material formation,^44,48^ and to control bacteria inside multicellular organisms.^34^ A palette of setups for light-regulated expression in bacteria have become available that jointly provide sensitivity to different wavelengths within the near-UV to near-IR regime. These setups can either involve second messengers (such as cyclic nucleotides),^64^ two-component systems (TCSs), or light-dependent protein–protein interactions. The interaction-based systems, mostly responsive to blue light, offer a more compact architecture and are frequently realized as a single polypeptide component.^29−32,65^ Although TCSs are larger and more complex by comparison, they have dominated optogenetic applications in bacteria. In part, the prevalence of light-regulated TCSs can be ascribed to their earlier availability,^8,18,20^ but it is also arguably due to the stringent responses many of these systems elicit as a function of light. Key to the ongoing use of TCSs is the ability of many histidine kinases to not only act as a kinase on their response regulator proteins but also as a phosphatase.^16^ The phosphorylation status and hence the activity of the RR can be modulated bimodally and stringently. By contrast, systems involving light-triggered changes in oligomerization or dimerization reactions as part of their regulatory mechanism may display pronounced dependence on protein concentration. Their light-dependent responses are largely governed by the laws of mass action and may consequently exhibit comparatively shallow activation profiles.^68^

Against this backdrop, we reprogrammed the pDusk and pDawn plasmids, among the most widely used setups for the optogenetic regulation of bacterial gene expression,^28,43−48^ from blue-light to red-light control. Doing so entailed the exchange of a blue-light-sensitive LOV photosensor domain by the PSM of the *D. radiodurans* bacteriophytochrome (Figure 1). Supply of the biliverdin chromophore, required by BphPs for absorption of red and far-red light, was ensured by inclusion of the *D. radiodurans* heme oxygenase. Upon transformation into *E. coli*, the resulting pREDusk and pREDawn plasmids mediated the downregulation and upregulation, respectively, of target gene expression under red light compared to darkness. Notably, pREDusk and pREDawn assemble all components required for light responses on a single, compact plasmid backbone, thus rendering them portable, versatile, and straightforward to use. Both plasmids exhibited high light sensitivity (with half-maximal responses at average light intensities of around 1–2 μW cm^–2^) and afforded stringent regulatory responses (Figures 1 and 2). In the case of pREDusk, the dynamic range (defined as the ratio of expression under dark and red-light conditions) amounted to around 200-fold and thereby much exceeded that of the parental pDusk system. Unlike the LOV-based parental systems, the light responses of pREDusk and pREDawn could be counteracted by another wavelength, namely far-red light (Figure 3), which equips the systems with an additional layer of control.

Sensitivity to red light, as opposed to blue light in pDusk and pDawn, provides at least two decisive advantages. First, as most setups for light-regulated bacterial expression respond to shorter wavelengths, predominantly in the blue spectral region, pREDusk and pREDawn lend themselves to be orthogonally addressed and jointly used with these systems. We evaluated this concept by combining pREDusk and pREDawn with the pCrepusculo and pAurora plasmids^66^ that encode the LOV receptor PAL and downregulate and upregulate, respectively, the expression in response to blue light (Figure 5). As expected, based on the action spectra of LOV receptors,^11^ neither pCrepusculo nor pAurora was toggled by red light. Conversely, pREDusk and pREDawn were not activated by blue light at the intensities and wavelengths currently used, which was unexpected given that the Soret band of *Dr*BphP partially absorbs at the illumination wavelengths^69^ (Figure S5b). Moreover, other receptors based on *Dr*PSM could be activated by blue light.^57^ The absent (or, at most weak) response of pREDusk and pREDawn to blue light can be exploited for multiplexing with diverse setups for the optogenetic control of gene expression or other cellular processes. Such approaches appear straightforward because pREDusk and pREDawn are self-contained and can be readily used as is without requiring any modification. Alternatively, the underlying light-sensitive *Dr*F1/FixJ TCS may also be integrated into optogenetic circuits with other photoreceptors. As a case in point, the PAL receptor not only responds to blue light (rather than red light) but also acts at the RNA level (rather than at the DNA level), thus making it particularly attractive in this regard. Irrespective of the precise approach, multiplexed optogenetic control stands to benefit, for example, biotechnological production processes and the manufacture of spatially patterned materials.^43,44,48^

Second, red and far-red light have superior penetration through biological tissues, while at the same time exhibiting reduced phototoxicity.^33^ As pREDusk and pREDawn both possess high sensitivity to red light, they may prospectively drive applications in tissue-embedded bacteria. Candidate application scenarios include the modulation of the gut microbiome and the use of custom-tailored microorganisms as novel therapeutics.^70^ Although the successful implementation of such applications will doubtless require substantial efforts, we probed their fundamental feasibility. We investigated the actuation of pREDawn through materials that mimic the optical properties of mouse skull and skin, respectively (Figure 6). Nearly unimpaired activation was achieved at therapeutically safe light intensities even when the applied light passed through materials with the optical properties of up to 1 mm mouse skull. These data underline the principal validity of red-light-dependent optogenetic applications inside multicellular organisms. It is worth noting that the pDawn plasmid has recently been employed in a similar fashion inside the mouse intestine.^34^ As blue light, necessary for pDawn activation, is too strongly absorbed and scattered by biological tissues, the study required the use of infrared radiation and upconverting nanoparticles, which, however, rendered the approach no longer fully genetically encodable. The pREDawn system, established at present, may bring an immediate benefit and thus supplant pDawn for this and related applications.

Compared to blue-light-sensitive systems, optogenetic circuits that control bacterial expression in response to red light possess at least two traits that may prove disadvantageous for application. First, as is true for pREDusk and pREDawn, sensitivity to red light is commonly achieved via sensory photoreceptors of the phytochrome superfamily. As noted above, these receptors require bilin chromophores which do not generally occur in bacteria and hence need be supplied. Within pREDusk and pREDawn, we met this challenge by including a heme oxygenase enzyme within a single operon together with the *Dr*F1:FixJ TCS. We therefore built self-contained systems that integrate all required components within a single, compact, and portable cassette. Second, although phytochromes maximally respond to red and far-red light, they also absorb to certain extent lower wavelengths (see Figure S5). At high intensity, blue light is thus expected to also activate phytochrome-based circuits to substantial degree, as indeed borne out in another study.^57^ While exhibiting high sensitivity to red light, pREDusk and pREDawn are, however, relatively insensitive to blue light and thereby surmount this challenge. Owing to this favorable property, we could trigger blue-light-responsive genetic circuits to full extent without inadvertent co-activation of pREDusk and pREDawn included in the same bacterial cell (see Figure 5). Although not probed here, we however expect that very strong blue light, more than used at present, eventually triggers pREDusk and pREDawn.

### Bacteriophytochrome Mechanism and Design

Beyond establishing new avenues for optogenetic control of bacterial expression, our work also sheds light on the signaling mechanism and the design of bacteriophytochrome receptors. Both the pREDusk and pREDawn plasmids harness the chimeric *Dr*F1 histidine kinase that we constructed by connecting the *Dr*PSM to the HK effector of FixL (see Figure 1a). Data acquired on pREDusk in darkness and under red light indicate that *Dr*F1 is a functional enzyme and that its net kinase activity is repressed by red light, akin to the repression of YF1 net kinase activity by blue light.^23^ Notably, these findings affirm our earlier observation that the *Dr*PSM is generally capable of regulating kinase activity, although wild-type *Dr*BphP exclusively acts as a net phosphatase.^41^ Put another way, a single structural framework and its light-dependent conformational changes suffice for the regulation of both the elementary kinase and phosphatase activities.^71^ Moreover, the successful and ready design of *Dr*F1 reveals considerable commutability of sensor and effector modules among bacteriophytochromes, LOV receptors, and sensor histidine kinases. At least subsets of these receptor families apparently harness highly similar or at least compatible signaling strategies, despite the disparate structure.^16^ In a similar vein, these mechanistic commonalities likely extend to a diverse and large group of modular signal receptors in nature which arguably arose during evolution via the recombination of much smaller sets of sensor and effector blocks.^72^

As the pREDusk setup provides a fast, if indirect, readout on the activity and light response of the underlying histidine kinase, it can serve to efficiently screen and analyze in depth sizeable numbers of receptor variants. In this way, the *Dr*PSM could, for instance, be exchanged for PSMs from different BphPs including from bathy phytochromes that adopt the Pfr form as their dark-adapted state. Similarly, the *Dr*PSM could be varied rationally or randomly, in particular within its chromophore-binding pocket and the PHY tongue, both of which determine how chromophore Z/E isomerization channels into downstream responses.^71^ When constructing *Dr*F1, we maintained the same linker spacing between the sensor and effector entities as in the wild-type *Dr*BphP which also bears a HK effector (see Figure 1a). Work on YF1 and other receptors had, however, revealed that receptor activity and response to signal can strongly depend on the length and, to lesser extent, the sequence of the linker intervening the sensor and effector modules.^23,54,57,73^ Elongation or shortening of this linker sufficed to drastically alter the system output of certain receptors, for example, to convert the blue-light-repressed YF1 into a variant whose net kinase activity was enhanced by blue light.^73^ The pREDusk plasmid constitutes an efficient screening platform that will allow the systematic mapping of linker variations in bacteriophytochrome histidine kinases. Doing so not only stands to yield insight into the molecular mechanism of signal transduction but augurs red-light-regulated HK variants with divergent and enhanced properties that can be harnessed for optogenetics.

## Methods

### Cloning and DNA Material

The phytochrome gene from *D. radiodurans* strain R1 (DrBphP, gene DR_A0050) in the pET-21b(+) plasmid (Novagen) was a kind gift from Prof. Richard Vierstra.^74,75^ The pDusk-*Ds*Red and pDawn-*Ds*Red plasmids^18^ were used to construct pREDusk and pREDawn, respectively. The gene fragment encoding *Dr*HO and the *Dr*BphP PCM was amplified from *D. radiodurans* genomic DNA (strain DSMZ-20359, Deutsche Sammlung für Mikroorganismen und Zellkulturen). Plasmid derivatives were cloned by using the NEBuilder HiFi DNA assembly cloning kit (New England Biolabs). For pREDusk, the *B. subtilis* YtvA LOV domain was replaced with the PAS-GAF-PHY fragment of DrBphP (residues 1–506). In addition, the HO gene (*Dr*HO) was introduced before *Dr*BphP, thus recapitulating the HO-bphP operon structure in the *D. radiodurans* genome. For cloning pREDawn, a gene-inversion cassette based on the λ phage cI inverter was amplified from pDawn and introduced into pREDusk downstream of the FixK2 promoter, like in ref (18). To facilitate inclusion of other target genes, pREDusk and pREDawn variants were generated where the *Ds*Red gene is replaced by a multiple-cloning site (MCS) derived from the pET-28c plasmid.^18^ For combination with the pCrepusculo and pAurora systems, the YPet (Nguyen et al., 2005) gene was synthesized with *E. coli*-adapted codon usage (GeneArt, ThermoFisher, Regensburg, Germany) and cloned into both pREDusk and pREDawn via Gibson assembly.

For the streptomycin-resistant constructs, the KanR gene and ORI were replaced with the StrR gene and CDF origin of replication from a pCDF-Duet plasmid (Novagen). To generate the ampicillin-resistant versions of the constructs, the KanR gene was replaced with an AmpR gene and its promoter from the pET-21b(+) plasmid (Novagen). The construct sequences were confirmed by sanger sequencing (Eurofins Genomics, Germany). All plasmids with multiple cloning cite (MCS) and their maps are available in Addgene (see section Accession Codes).

### Analysis of Light–Dose Response

To assess the response of the pREDusk and pREDawn systems to different illumination regimes, the pREDusk-*Ds*Red and pREDawn-*Ds*Red plasmids were transformed into the *E. coli* CmpX13 strain^76^ which was used for the development and characterization of the parental pDusk and pDawn systems.^18^ 5 mL lysogeny broth (LB) medium was supplemented with 50 μg μL^–1^ kanamycin (Kan) and inoculated with CmpX13 cells harboring pREDusk-*Ds*Red or pREDawn-*Ds*Red. The cultures were incubated for 24 h at 30 °C and 225 rpm agitation under non-inducing conditions (i.e., 100 μW cm^–2^ 660 nm light for pREDusk or darkness for pREDawn). Following 100-fold dilution in fresh LB/Kan medium, 200 μL of each of the cell suspension was dispensed into individual wells of 96-well clear-bottom, black-walled microtiter plates (μClear plates, Greiner BioOne, Frickenhausen, Germany). Plates were sealed with a gas-permeable film (BF-410400-S, Corning, New York, USA) and placed on top of a programmable matrix equipped with an 8 × 8 array of three-color LEDs at emission wavelengths of (463 ± 12) nm, (521 ± 14) nm, and (624 ± 8) nm.^62,77^ To assess the influence of simultaneously applied or interleaved red/far-red light, a custom-made LED array configured with (660 ± 10) nm and (850 ± 21) nm LEDs was used instead.^56^ In either case, using an Arduino microcontroller, the illumination intensity and timing can be configured for each of 8 × 8 wells individually. Unless stated otherwise, all experiments used a 1:10 duty cycle in which samples were repeatedly illuminated for 20 s, followed by 180 s darkness. Light intensities of the LED array were calibrated with a power meter (model 842-PE equipped with a 918D-UV-OD3 silicon detector, Newport, Darmstadt, Germany). The sealed MTPs were incubated for 18 h at 37 °C inside an incubator (HN-2 Herp Nursery II, Lucky Reptile, Waldkirch, Germany) while being agitated at 750 rpm (PMS-1000i shaker, Grant instruments, Cambridge, United Kingdom). The optical density at 600 nm (OD_600_) and *Ds*Red fluorescence (F) of the cultures were measured with a Tecan Infinite M200 PRO multimode MTP reader (Tecan Group, Ltd., Männedorf, Switzerland). For fluorescence measurements, excitation and emission wavelengths were set to (554 ± 9) nm and (591 ± 20) nm, respectively. Fluorescence readings were normalized to OD_600_ and are reported as mean ± s.d. of three biological replicates. Data were corrected for background fluorescence (determined for bacteria harboring the pREDusk-MCS negative control), plotted, and evaluated with the Fit-o-mat software.^78^ Dose–response data acquired at different light intensities I were fitted to Hill binding isotherms
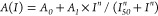
1where *n* is the Hill coefficient and *I*_50_ is the light intensity at half-maximal response.

The combination of the red-light-responsive pREDusk-YPet and pREDawn-YPet with the blue-light-responsive pCrepusculo and pAurora setups^66^ was studied likewise with the following modifications to the protocol. To address the systems separately, the pCrepusculo and pAurora systems were equipped with the *Ds*Red fluorescent reporter. CmpX13 cells were transformed with a combination of pREDusk-YPet/pREDawn-Ypet and pCrepusculo-*Ds*Red/pAurora-*Ds*Red. The resultant cultures were cultivated in LB medium supplemented with 50 μg μL^–1^ Kan and 100 μg μL^–1^ streptomycin (Strep). During growth in sealed MTPs, cultures were illuminated from below with blue (463 ± 12) nm and/or red light (624 ± 8) nm. Blue and red light were applied simultaneously using a 1:10 duty cycle. YPet fluorescence was measured at (500 ± 9) nm excitation and (530 ± 20) nm emission. Cross-talk between the YPet and *Ds*Red fluorescence channels was below 0.1%. Normalized and averaged *F*/OD_600_ values for *Ds*Red and YPet were plotted using Python/matplolib.

### Light Induction Kinetics

Bacteria harboring pREDusk-*Ds*Red or pREDawn-*Ds*Red were cultivated in 5 mL LB/Kan medium for 16–18 h at 37 °C and 225 rpm shaking under non-inducing conditions (i.e., 100 μW cm^–2^ 660 nm light for pREDusk and darkness for pREDawn). The cultures were then used to inoculate 100 mL LB/Kan medium in a baffled Erlenmeyer flask, and incubation was continued at 37 °C and 225 rpm shaking. At an OD_600_ of 0.5, the cultures were transferred to inducing conditions (i.e., darkness for pREDusk and 100 μW cm^–2^ 660 nm light for pREDawn) and incubation was continued. Samples of 200 μL were drawn at the time point of induction and after. To arrest cell growth and translation, the aliquots were supplemented with 3.5 mg mL^–1^ chloramphenicol and 0.4 mg mL^–1^ tetracycline.^18^ Following ≥2 h incubation to allow *Ds*Red maturation (Figure S2b),^49^ OD_600_ and fluorescence were determined for three biological replicates as described above. The normalized and averaged fluorescence values were plotted against time since induction and fitted to a generalized logistic function [eq 2]^18,78^ to determine the time point *t*_50_ for half-maximal induction.
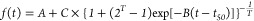
2

The parameters *A* and *C* denote the background fluorescence and the normalized fluorescence of cultures in the stationary phase at longer incubation times, respectively. The parameters *B* and *T* determine the shape of the logistic function and hold no readily interpretable physical meaning.

### Flow Cytometry

Bacterial cultures of 100 mL were inoculated with single bacterial clones from plates grown under non-inducing conditions overnight bearing either pREDusk-*Ds*Red or pREDawn-*Ds*Red. Cultures were then incubated at 37 °C and 220 rpm agitation under non-inducing conditions (i.e., 100 μW cm^–2^ 660 nm light for pREDusk or darkness for pREDawn) until they reached an OD_600_ of 0.5. The cultures were then transferred to inducing conditions (i.e., darkness for pREDusk or 100 μW cm^–2^ 660 nm light for pREDawn). Control cultures bearing pREDusk-MCS were grown under non-inducing conditions. After 20 h of induction, 200 μL samples were taken from the cultures, treated with antibiotics (3.5 mg mL^–1^ chloramphenicol and 0.4 mg mL^–1^ tetracycline), and incubated on ice for 2 h to allow *Ds*Red maturation (see above). For flow cytometry, the bacteria were pelleted by centrifugation (1845 rcf for 2.5 min) and resuspended in 500 μL of PBS (0.14 M NaCl, 0.0027 M KCl, and 0.01 M phosphate buffer, pH 7.4). The samples were then diluted 1:10 in PBS and kept on ice in dark until data acquisition. Flow cytometry was performed on a NovoCyte Quanteon 4025 instrument using a 561 nm excitation laser and a (586 ± 20) nm bandpass emission filter. Approximately 100,000 cells were collected for three biological replicates in three separate runs. The FCS data were sorted using a Python script, in which the lower threshold value for forward and side scatter was set to 100. Data were fitted to a skewed Gaussian probability density function as implemented in the Python module script.^78^

### Bacterial Photography

To generate bacteria for photography, bacteria bearing the pREDawn-*Ds*Red plasmid were cultured in 5 mL LB/Kan for 16–18 h in darkness (37 °C, 225 rpm agitation). 1 mL of the culture was then rapidly mixed with 9 mL of liquid LB/Kan medium containing 1.4% (w/v) agar. The solution was poured into Petri dishes and allowed to solidify, followed by incubation for 2 h at 37 °C in darkness. An image of a star was printed on a transparent film (Laserfilm CGF 640, Pelikan) with a conventional laser printer. Using a Lambda Mini Evo 640–75 (RGB Lasersystems, Kelheim, Germany) laser with the emission of 640 nm at ∼200 μW cm^–2^ emitted power, the image was projected onto the plate for 5 min (Figure 4a). After illumination, the bacterial plates were incubated at 37 °C for 16–18 h. Fluorescence was recorded on a transilluminator equipped with 470 nm LEDs and an amber emission filter (FG-08, Nippon Genetics Europe). Photographs were taken with an Omegon VeLOX 178C CMOS camera.

### Fluorescence Microscopy

Bacteria bearing pREDawn-*Ds*Red were cultured on LB/Kan plates in dark for 16–18 h. Then, a 5 ml LB culture was started from the plate and grown in dark for 16–18 h. One hour before the microscopy imaging, 1.4% (w/v) agar slabs containing bacteria were prepared like in ref (79). Two microscope slides were placed side-by-side 5 mm apart on top of a third microscope slide. The resulting chamber was filled with ∼1 mL of liquid LB-agar and covered with yet another microscope slide. The slab was let to solidify for 30 min. Then, 2 μL of 1:100 cell culture dilution was added on a 35 mm Petri dish with a 1.5 mm coverglass bottom (MatTek Corporation). The bacteria were covered with a 2 × 2 mm agar slab and a thin microscope cover slide to prevent drying and kept in dark until microscopy. For imaging, a fully motorized Nikon Eclipse Ti-E inverted widefield microscope was used with an environmental chamber set to +37 °C. The sample position was searched under transmitted light with a green 546 nm filter, followed by 15 min illumination with a 780 nm LED light (∼100 μW cm^–2^). To activate the selected region of pREDawn-*Ds*Red cells, a region of interest was illuminated for 1 h with pulsed red light (640 nm, 240 mW cm^–2^), where 100 ms pulses were applied at 1 min intervals. The illumination period was followed by 1 h recovery time in the dark, after which the fluorescence images were acquired at 30 min intervals to visualize the *Ds*Red fluorescence (ex. 549/em. 600).

### Illumination through Tissue Phantoms

To gauge the principal potential for applying pREDawn and related systems inside mammalian hosts, we prepared disc-shaped tissue phantoms of 4.5 cm diameter. Individual phantoms mimic the optical properties of 300 or 1000 μm thick mouse skin or skull in the wavelength range 600–900 nm. The phantom discs were 3D-printed using stereolithography with a UV (405 nm)-curing polymer resin (Elastic 50A, Formlabs Inc). The resins yielded a transparent and elastic matrix for embedding of the phantom components. A scattering component was introduced by adding ZnO nanoparticles with an average diameter of 340 nm. The scattering level was tuned to yield reduced scattering coefficients of μ_s_’ = 0.8 mm^–1^ for the mouse skin^80^ and μ_s_’ = 3.3 mm^–1^ for the mouse skull^81^ at a wavelength of 650 nm. An absorption component was introduced by adding a black color pigment to provide an absorption coefficient μ_a_ of 0.15 mm^–1^ in the considered spectral range of 600–900 nm. Scattering/absorption parameters of the phantom layers were determined spectrophotometrically with integrating spheres (Optronic Laboratories, USA).^82,83^

For experiments with pREDawn, we first assessed the transmission through the phantom discs using 660 nm LEDs and a SEC2020 UV/vis spectrophotometer (ALS Co. Ltd, Tokyo, Japan). Next, 5 mL LB/Kan cultures of bacteria harboring pREDawn-*Ds*Red were grown for 16–18 h at 37 °C and 225 rpm shaking in darkness. The cultures were then diluted 10^6^-fold in LB and plated on LB/Kan agar. Following incubation for 16–18 h at 37 °C in darkness, plates were illuminated with 660 nm light (50 and then 5 μW cm^–2^) for up to 120 min, either without or with one of the discs in the light path. After illumination, plates were further incubated for 18 h at 37 °C in darkness to allow the expression and maturation of *Ds*Red. A single bacterial colony was picked and dissolved in H_2_O. OD_600_ and *Ds*Red fluorescence were measured and normalized as described above. Data represent mean ± s.d. of three biological replicates.

## Supporting Information Available

Supplementary experiments with the pREDusk and pREDawn tools and their variants with different antibiotic resistance, effects of illumination on the systems and bacteria, and absorption/emission spectra related to the study (PDF)
Experimental data from Multamäki et al. (2022) (XLSX)

## Accession Codes

MCS versions of the tools have been deposited at Addgene repository (http://www.addgene.org). Accession numbers: 188970 (pREDusk-MCS), 188971 (pREDawn-MCS), 188972 (pREDusk-StrR-MCS), 188979 (pREDawn-StrR-MCS), 188974 (pREDusk-AmpR-MCS), and 188978 (pREDawn-AmpR-MCS).
