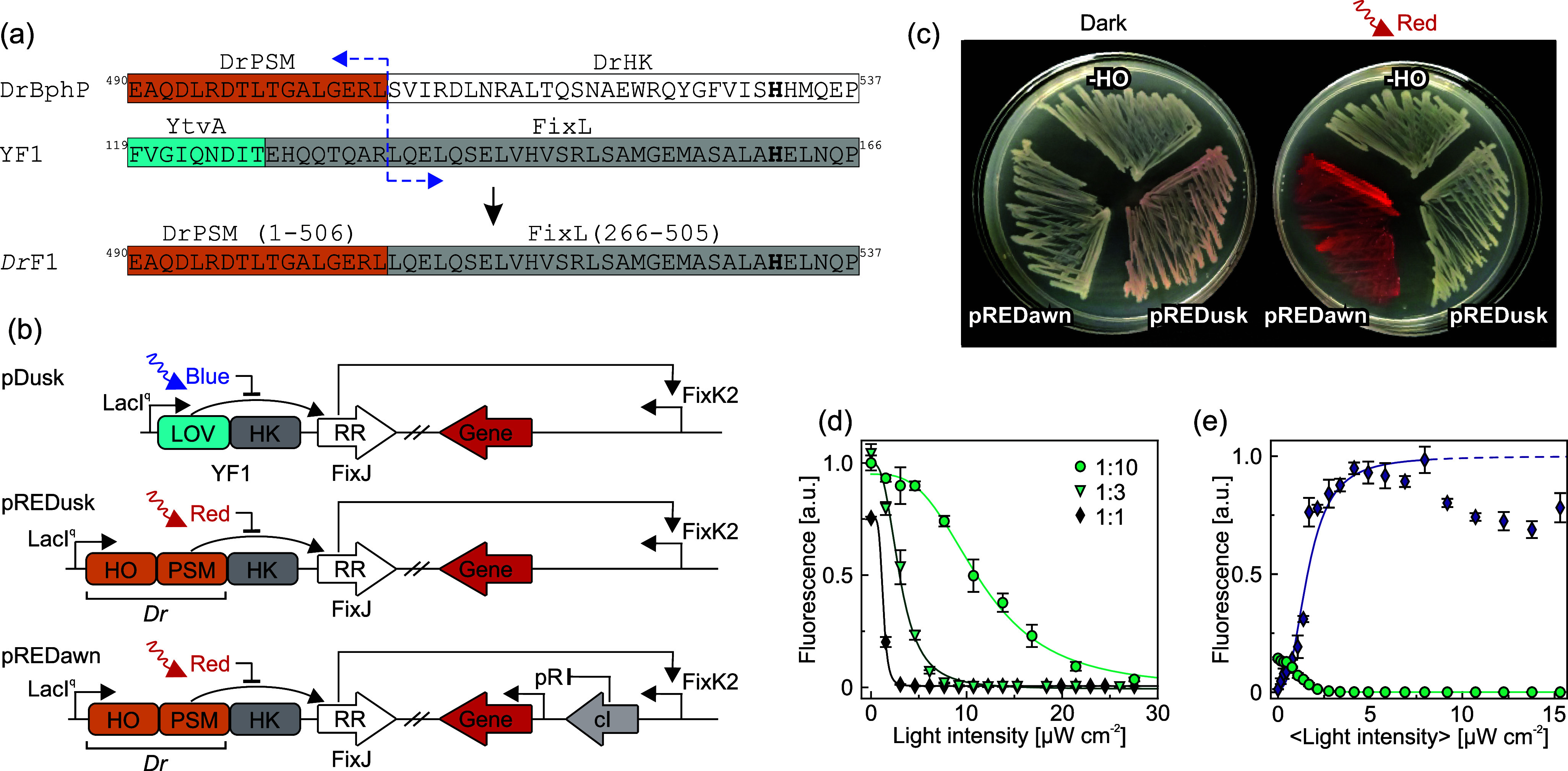# Correction to “Optogenetic Control of Bacterial Expression by Red Light”

**DOI:** 10.1021/acssynbio.4c00601

**Published:** 2024-09-18

**Authors:** Elina Multamäki, Andrés García de Fuentes, Oleksii Sieryi, Alexander Bykov, Uwe Gerken, Américo Tavares Ranzani, Jürgen Köhler, Igor Meglinski, Andreas Möglich, Heikki Takala

**Affiliations:** † Department of Anatomy, University of Helsinki, 00014 Helsinki, Finland; ‡ Lehrstuhl für Biochemie, Photobiochemie, Universität Bayreuth, 95447 Bayreuth, Germany; § Optoelectronics and Measurement Techniques, University of Oulu, 90014 Oulu, Finland; ∥ Lehrstuhl für Spektroskopie Weicher Materie, Universität Bayreuth, 95447 Bayreuth, Germany; ⊥ College of Engineering and Physical Sciences, Aston University, B4 7ET Birmingham, United Kingdom; # Department of Biological and Environmental Science, Nanoscience Center, University of Jyvaskyla, 40014 Jyvaskyla, Finland

An error was found in panel a of Figure 1 of the publication entitled “Optogenetic Control of Bacterial Expression by Red Light”. There, a text element after “*Dr*F1” is accidentally shifted by one letter to the right in relation to the rest of the figure. Here, we provide a corrected version of Figure 1.

**Figure 1:**