# Supplementary material for: Optogenetic Control of Bacterial Expression by Red Light
Source: ACS Synth Biol. 2022 Aug 23;11(10):3354–67. doi: 10.1021/acssynbio.2c00259 (PMC9594775; doi:10.1021/acssynbio.2c00259)
Supplement: Supplementary file 1 — sb2c00259_si_001.pdf [file sb2c00259_si_001.pdf]

Optogenetic Control of Bacterial Expression by Red Light

Elina Multamäki[1,#], Andrés García de Fuentes[2,#], Oleksii Sieryi[3], Alexander Bykov[3], Uwe Gerken[4], Américo Tavares Ranzani[2], Jürgen Köhler[4], Igor Meglinski[3,5], Andreas Möglich[2,*], and Heikki Takala[1,6,*]

[1] Department of Anatomy, University of Helsinki, 00014 Helsinki, Finland
[2] Lehrstuhl für Biochemie, Photobiochemie, Universität Bayreuth, 95447 Bayreuth, Germany
[3] Optoelectronics and Measurement Techniques, University of Oulu, 90014 Oulu, Finland
[4] Lehrstuhl für Spektroskopie weicher Materie, Universität Bayreuth, 95447 Bayreuth, Germany
[5] College of Engineering and Physical Sciences, Aston University, B4 7ET, Birmingham, UK
[6] Department of Biological and Environmental Science, Nanoscience Center, University of Jyvaskyla, 40014 Jyvaskyla, Finland

[#] These authors contributed equally
* To whom correspondence should be addressed: H.T. heikki.p.takala@jyu.fi, +358 46 923 6211; A.M. andreas.moeglich@uni-bayreuth.de, +49 921 55 7835

SUPPORTING INFORMATION

Supporting information includes Supporting Figures (1–5), Supporting Table 1, and Supporting References.

TABLE OF CONTENTS

SUPPORTING FIGURE 1 2
SUPPORTING FIGURE 2 3
SUPPORTING FIGURE 3 4
SUPPORTING FIGURE 4 5
SUPPORTING FIGURE 5 6
SUPPORTING TABLE 1 7
SUPPORTING REFERENCES 8

SUPPORTING FIGURE 1

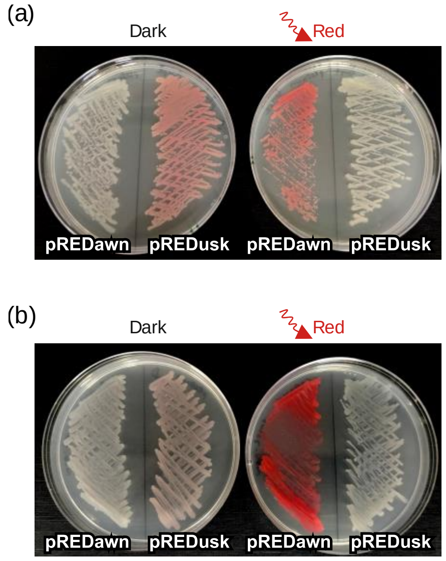

Supporting Figure 1. Bacterial plates with alternative antibiotic resistance versions of pREDusk and pREDawn. (a) The streptomycin-resistant versions pREDusk-StrR and pREDawn-StrR. (b) The ampicillin-resistant versions pREDusk-AmpR and pREDawn-AmpR. In the pREDawn versions, the expression of the *Ds*Red fluorescent reporter is activated with red light, and in the pREDusk versions, *Ds*Red expression is diminished under red light. See Table 1 for full list of alternative constructs.

SUPPORTING FIGURE 2

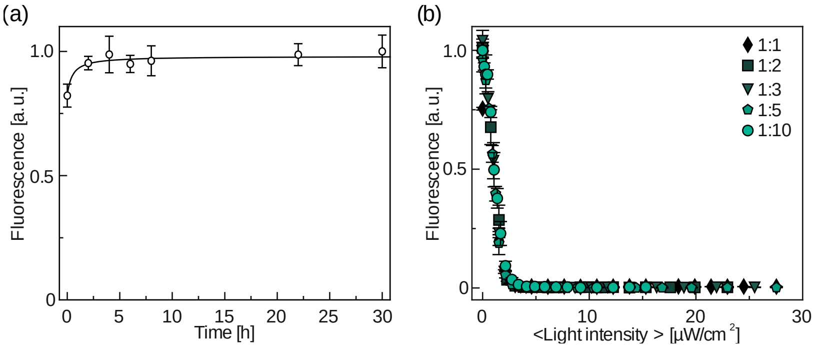

Supporting Figure 2. The stability of *Ds*Red over time and the effect of discontinuous illumination on the output of pREDusk. (a) To assess *Ds*Red stability over time, bacteria carrying the pREDusk-*Ds*Red plasmid were grown at 37°C and 220 rpm under inducing conditions (i.e., darkness) to an OD_{600} of around 0.75. Then, cell growth and expression were halted with antibiotics (3.5 mg mL^{-1} chloramphenicol and 0.4 mg mL^{-1} tetracycline), and the *Ds*Red fluorescence was monitored over time with Victor X3 (Perkin Elmer). (b) Triggering of pREDusk-*Ds*Red by discontinuous illumination. Bacteria harboring the pREDusk-*Ds*Red plasmid were cultivated while being exposed to intermittent red light at varying intensities and at duty cycles of 1:1 (diamonds), 1:2 (squares), 1:3 (triangles), 1:5 (pentagons), or 1:10 (circles). Following incubation, the *Ds*Red reporter fluorescence in the cultures was normalized by the optical density of the cultures at 600 nm (OD_{600}). The data are plotted as a function of time-averaged light intensity.

SUPPORTING FIGURE 3

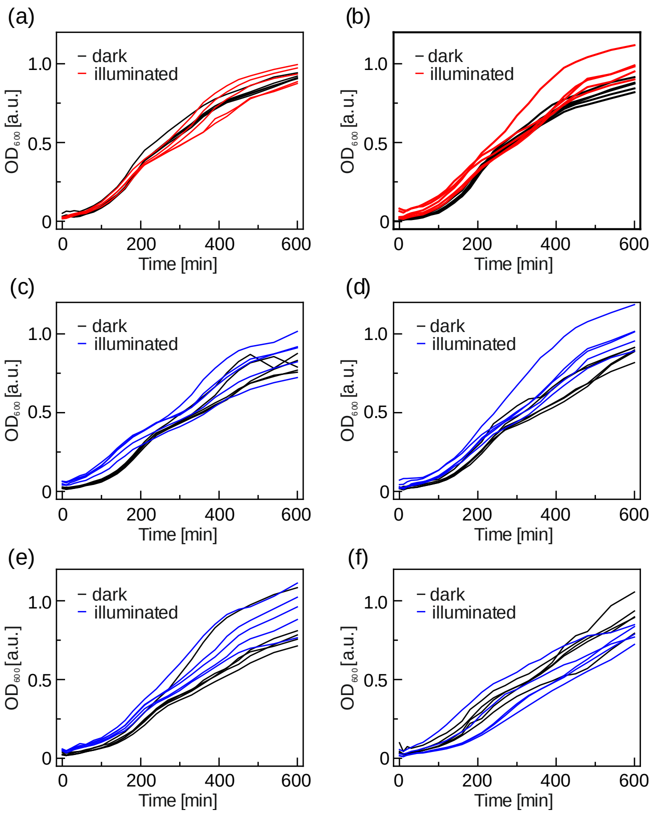

Supporting Figure 3. The effect of illumination on the growth of bacterial cultures harboring (a) pREDusk-*Ds*Red, (b) pREDawn-*Ds*Red, (c) pDusk-*Ds*Red, (d) pDawn-*Ds*Red, (e) pCrepusculo-*Ds*Red, and (f) pAurora-*Ds*Red. For each plasmid, five saturated overnight cultures (biological replicates) were used to inoculate 5 × 200 µL of LB/Kan medium in microtiter plates. The plates were incubated at 37°C while shaking, and the optical density at 600 nm (OD_{600}) was measured periodically. The black lines originate from plates incubated in darkness; the colored lines are from plates incubated under light (color according to light source used). With a 1:10 duty cycle, the average light intensity was 3.1 µW cm^{-2} for red light and 8.4 µW cm^{-2} for blue light. These values were selected based on the multiplexing experiments shown in Figure 5.

SUPPORTING FIGURE 4

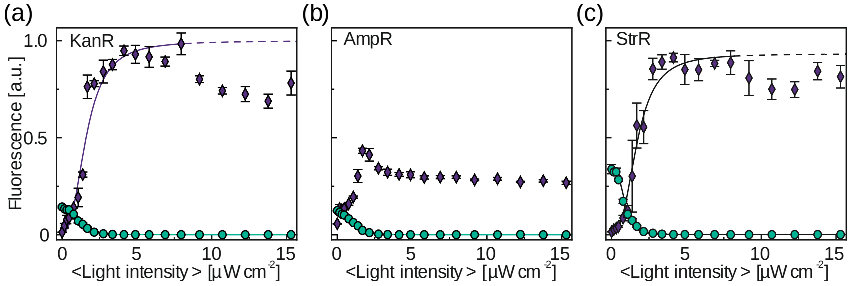

Supporting Figure 4. The response to red light of bacteria harboring pREDusk (circles) and pREDawn (diamonds) with different antibiotic resistances, acquired under 1:10 duty cycle. The antibiotic resistances are: (a) kanamycin (KanR), (b) ampicillin (AmpR), and (c) streptomycin (StrR). The mean fluorescence ± s.d. of three biological replicates is shown. All values are normalized to the maximum fluorescence value obtained for the kanamycin-resistant pREDawn under red light (panel a, diamonds). The data in panel a are the same as in Figure 1e and are included for direct comparison.

SUPPORTING FIGURE 5

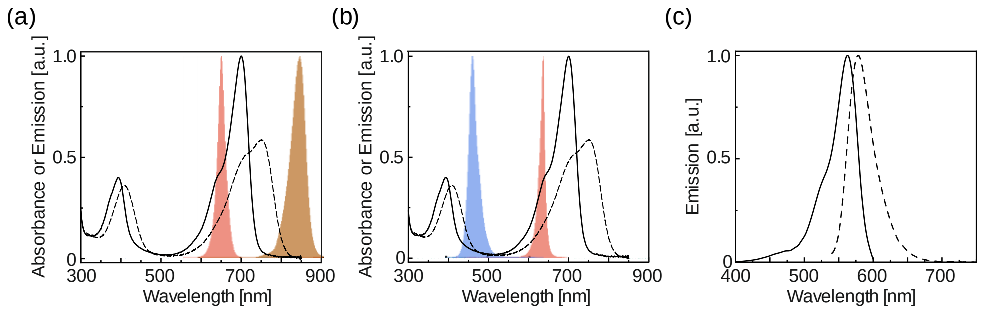

Supporting Figure 5. Emission spectra of selected light sources, absorption spectra of the *Dr*PSM in dark (solid line) and after 655-nm light exposure (dashed line), and *Ds*Red fluorescence spectra. Absorption spectra of the *Dr*PSM are adapted from [1]. (a) The stack plots show the emission spectra of the red and far-red LEDs that were applied to the characterization of pREDusk and pREDawn (see Figure 3) [2]. (b) The emission spectra of the blue and red LEDs [3] used for studying the combined light responses of pREDusk/pREDawn and pCrepusculo/pAurora (see Figure 5). (c) The fluorescence excitation (solid line) and emission spectra of *Ds*Red (dotted line), adapted from [4].

SUPPORTING TABLE 1

**Phantom type**	**Optical thickness [µm]**	**Light intensity [µW cm^{-2}]**	**Rate of fluorescence increase [min^{-1}] [a]**
none	n/a	5	$(4.7 \pm 0.4) \times 10^{-3}$
		50	$(5.3 \pm 0.8) \times 10^{-3}$
skin	300	5	$(3.9 \pm 0.6) \times 10^{-3}$
		50	$(3.2 \pm 0.3) \times 10^{-3}$
	1000	5	$(3.4 \pm 0.6) \times 10^{-3}$
		50	$(3.5 \pm 0.5) \times 10^{-3}$
skull	300	5	$(3.6 \pm 0.2) \times 10^{-3}$
		50	$(4.1 \pm 0.6) \times 10^{-3}$
	1000	5	$(1.9 \pm 0.2) \times 10^{-3}$
		50	$(4.6 \pm 0.6) \times 10^{-3}$

[a] The values refer to Figure 6 and denote by how much the normalized *Ds*Red reporter gene fluorescence increased per minute of illumination with 660-nm light.

SUPPORTING REFERENCES

1. Takala, H., Lehtivuori, H. K., Berntsson, O., Hughes, A., Nanekar, R., Niebling, S., Panman, M., Henry, L., Menzel, A., Westenhoff, S., and Ihalainen, J. A. (2018) On the (un)coupling of the chromophore, tongue interactions, and overall conformation in a bacterial phytochrome. *J. Biol. Chem. 293*. 8161-8172.
2. Stüven, B., Stabel, R., Ohlendorf, R., Beck, J., Schubert, R., and Möglich, A. (2019) Characterization and engineering of photoactivated adenylyl cyclases *J. Biol. Chem. 400*. 429-441.
3. Hennemann, J., Iwasaki, R. S., Grund, T. N., Diensthuber, R. P., Richter, F., and Möglich, A. (2018) Optogenetic Control by Pulsed Illumination. *ChemBioChem 19*. 1296-1304.
4. Golonka, D., Gerken, U., Köhler, J., and Möglich, A. (2020) The Association Kinetics Encode the Light Dependence of Arabidopsis Phytochrome B Interactions. *J. Mol. Biol. 432*. 4327-4340.
